# Effectiveness of winter temperatures for satisfying chilling requirements for reproductive budburst of red alder (*Alnus rubra)*

**DOI:** 10.7717/peerj.5221

**Published:** 2018-09-25

**Authors:** Janet S. Prevéy, Constance A. Harrington

**Affiliations:** Pacific Northwest Research Station, United States Department of Agriculture - Forest Service, Olympia, WA, United States of America

**Keywords:** Climate change, Flowering, Phenology

## Abstract

**Background:**

Experiencing an adequate amount of cold temperatures over winter is necessary for many temperate tree species to break dormancy and flower in spring. Thus, changes in winter and spring temperatures associated with climate change may influence when trees break dormancy and flower in the future. There have been several experimental studies that have quantified the effectiveness of cold temperatures for chilling requirements for vegetative budburst of temperate trees; however, there are few experimental studies addressing the chilling requirements for reproductive budburst of trees, as it is difficult to place reproductively mature trees in temperature-controlled environments.

**Methods:**

To identify how changing temperatures associated with climate change may impact reproductive phenology, we completed a temperature-controlled growth chamber experiment using cuttings of reproductive branches of red alder (*Alnus rubra),* one of the most widespread hardwood tree species of the Pacific Northwest, USA. The purpose of this study was to examine how colder (4 °C) and warmer (9 °C) winter temperature regimes influenced the timing of reproductive budburst of red alder cuttings in spring. We also compared the date of budburst of cuttings to that of branches from intact trees.

**Results:**

We found that cuttings flowered earlier after pretreatment with a 4 °C winter temperature regime than after a 9 °C winter temperature regime. We found no significant differences between the timing of male budburst of cuttings exposed to ambient conditions compared to male budburst of branches from intact trees. We used our experimental data to estimate a “possibility-line” that shows the accumulated chilling and forcing temperatures necessary prior to reproductive budburst of red alder.

**Discussion:**

This study provides a preliminary indication that warmer winters with climate change may not be as effective as colder winters for satisfying chilling temperature requirements of a Northwest hardwood tree species.

## Introduction

Tree phenology is strongly controlled by temperature, and as climate change alters seasonal temperatures, tree phenology may shift in unexpected ways (Luedeling, Zhang & Girvetz, 2009; Cook, Wolkovich & Parmesan, 2012). Trees in temperate regions have evolved to time spring phenological events so that they occur generally after the risk of frost has passed, and thus many tree species, or genotypes within species, require a certain period of cold (chilling) temperatures to break dormancy prior to flowering or leaf-out (Perry, 1971; Harrington, Gould & St.Clair, 2010). One of the most noticeable phenological changes over the recent past has been earlier leaf-out and flowering of tree species in temperate ecosystems (Fitter & Fitter, 2002; Parmesan & Yohe, 2003). However, continued warming, especially over winter, may result in a lack of chilling temperatures required for initiation of spring phenological events (Luedeling, Zhang & Girvetz, 2009), thus leading to a possible delay in spring phenology (Cook, Wolkovich & Parmesan, 2012). Additional research is needed on the specific chilling requirements of temperate tree species to enable prediction of how tree phenology, and associated changes in important ecosystem services, will change with climate change (Chuine et al., 2016).

There is a large body of literature that relies on observational data to estimate the chilling requirements for budburst of tree species (e.g., Hannerz, 1999; Chuine, 2000; Luedeling et al., 2009; Prevéy, Harrington & St. Clair, 2018). However, temperatures can fluctuate greatly over the winter, so it is difficult to quantify the effectiveness of specific temperatures for satisfying chilling requirements, and to separate the effects of temperature from other environmental cues under natural conditions. To address this, there have been a number of experimental studies that have examined the influence of simulated winter temperature regimes on vegetative budburst of small potted trees or twigs (e.g., Harrington, Gould & St.Clair, 2010; Basler & Körner, 2012; Nanninga et al., 2017). However, there have been few studies that examine how experimental winter temperatures influence the timing of reproductive budburst of trees, since it is difficult to place reproductively-mature trees in experimental treatments, such as growth chambers or greenhouses (but see Viti & Monteleone, 1995). One method to overcome this obstacle is to take cuttings (cut twigs) of reproductively mature trees, and place these cuttings in simulated temperature environments (Basler & Körner, 2012; Vitasse & Basler, 2014; Nanninga et  al., 2017; Flynn & Wolkovich, 2018).

From previous experimental studies, several patterns have emerged. Multiple studies indicate that exposure to increased cold, or chilling, temperatures reduces the amount of warm, or forcing, temperatures needed for reproductive budburst in spring (Harrington, Gould & St.Clair, 2010; Nanninga et al., 2017). Phenology models that assign different effectiveness values to cold and warm temperatures and sum the accumulations of these chilling and forcing units over the dormant season can be used to predict the timing of budburst in spring. A modeled “possibility-line” predicts the amount of forcing needed for reproductive budburst based on the amount of chilling a tree has received (Harrington, Gould & St.Clair, 2010; Prevéy, Harrington & St. Clair, 2018). However, phenology models vary in the accuracy of their predictions for different species (Chuine, Cour & Rousseau, 1999; Chuine et al., 2016). Thus, testing the equations of a reproductive phenology model with different species and under experimental conditions can help determine if it can be broadly applied to determine the timing of flowering of temperate tree species.

Here, we focus on the influence of winter temperature on flowering dates of red alder (*Alnus rubra*), the most common hardwood tree species of the Pacific Northwest (Harrington, 2006). Historically, red alder has received less research attention than some of the widespread conifer species of the region. However, more recently, the values of red alder as an important component of ecosystems and as a timber crop are being recognized, and thus more attention is being paid to this species (Deal & Harrington, 2006; Harrington, 2006). Red alder plays an important role in northwestern ecosystems by stabilizing streambanks, fixing nitrogen in soil, and providing food and cover for animals (Harrington, 2006; Harrington et al., 2008). Additionally, it has become a valuable timber species, and interest in the effects of management practices on tree growth, as well as flowering, has grown (Harrington & Debell, 1995; Ahrens & Bluhm, 2017). However, to date, there has been relatively little research on the environmental cues that are important for the reproductive phenology of red alder.

In the current study, we examine how experimental winter temperature regimes influence the date of reproductive budburst of cuttings of red alder. We created a range of experimental conditions in temperature-controlled growth chambers and greenhouses to address two questions: **(1)** How effective are relatively colder (4 °C) and warmer (9 °C) winter temperature regimes for chilling prior to reproductive budburst of red alder? Based on previous research (Sunley, Atkinson & Jones, 2006; Prevéy, Harrington & St. Clair, 2018), we hypothesized that temperatures at or below 5 °C would be more effective (or more quickly satisfy chilling requirements) than temperatures above 5 °C, so cuttings in treatments experiencing colder temperatures over winter would flower earlier than those experiencing warmer winters when exposed to forcing temperatures in spring. **(2)** Can a reproductive phenology model developed for reproductive budburst of Douglas-fir (Prevéy, Harrington & St. Clair, 2018) predict the accumulations of hourly chilling and forcing temperatures needed for reproductive budburst of red alder?

## Methods

### Sample collection and treatment

On November 1st 2016, we collected cuttings of red alder (*Alnus rubra*) from a riparian corridor along the edge of Webster Nursery, south of Olympia, WA (46°57′05.8″N, 122°57′50.8″W). All sampled trees were flagged so we could compare phenology of cuttings to phenology on intact trees in spring. We flagged branches of ten individual trees, and collected reproductive twigs from the branches of those ten individual trees. We placed the cut ends of twigs in water, and transported the twigs immediately to the USFS Olympia Forestry Sciences Laboratory, in Olympia, WA, where the experiment was conducted. On January 12th 2017, we collected an additional set of cuttings from seven of the ten originally sampled trees at Webster Nursery. The collection site is owned by the Washington State Department of Natural Resources, and they granted us permission to take plant samples from their property. Flowering usually occurs from mid-winter through early spring, with seed ripening from late August to October (Harrington et al., 2008).

### Cutting preparation

Prior to being placed in experimental treatments, all cuttings were recut to similar lengths (30–40 cm) and then the lower portions were submerged into a disinfectant sodium hypochlorite solution (200 ppm active chlorine) for ten seconds. They were then recut underwater and placed in containers filled with 400 ml water. The sides of all containers were covered in aluminum foil to block sunlight and reduce algal growth. Every seven days over the course of the experiment we changed the water in containers, recut the stems underwater, and randomized the location of containers in experimental treatments. We also recorded “survival” of cuttings each week. A cutting was considered dead if the cut stem was no longer green, or if the cutting had shed its reproductive buds. For the flagged branches of the sampled Webster trees, the reproductive buds of the branch were considered dead if they stop developing, and did not flower in spring. Portions of these methods were adapted from Basler & Körner (2012).

### Experimental treatments

We placed one cutting from each sampled tree (ten cuttings per treatment in total) in one of three different experimental treatments. The three treatments were: **4 °C**: a 4 °C temperature regime in a growth chamber starting on November 2nd 2016, **9 °C**: a 9 °C temperature regime in a growth chamber starting on November 2nd 2016, and **ambient/greenhouse**: ambient temperatures in a lathhouse starting on November 2nd 2016 (Table 1). We also had an **ambient** treatment where cuttings remained in a lathhouse over winter and spring. We compared the dates of reproductive budburst on the cuttings to the reproductive phenology on the flagged branches of intact trees at Webster Nursery to examine how phenology of cut branches from trees may differ from whole-tree phenology, which we will refer to here as the **Webster** treatment. Finally, we placed the additional set of cuttings sampled on January 12th 2017 from Webster Nursery in the greenhouse to increase the range of temperature conditions for modelling the possibility line of chilling and forcing conditions necessary to flower (**Webster/greenhouse** treatment, Table 1).

**Table 1:** The locations and average temperatures (°C) ±standard error for all experimental treatment combinations, and at Webster Nursery over the course of the experiment: November 2nd 2016 –March 30th 2017.

Treatment	November	December	January	February through budburst
4 °C	Constant 4 ± 0.009 °C			Greenhouse (ave. 16 ± 0.3 °C)
9 °C	Constant 9 ± 0.04 °C			Greenhouse (ave. 16 ± 0.3 °C)
Ambient/greenhouse	Variable temp. (ave. 4.4 ± 0.5 °C)			Greenhouse (ave. 16 ± 0.3 °C)
Ambient	Variable temp. (ave. 5.7 ± 0.43 °C)			
Webster/greenhouse	Variable temp. (ave. 4.3 ± 0.6 °C)		Greenhouse (ave. 14.4 ± 0.3 °C)	
Webster	Variable temp. (ave. 4.3 ± 0.4 °C)			

On January 31st 2017, cuttings from the **4 °C, 9 °C, ambient/greenhouse, and Webster/greenhouse treatments** were moved to a greenhouse with a variable temperature that averaged 16 °C to simulate forcing conditions. Temperature regimes for treatments were accomplished with a combination of growth chambers, ambient conditions in a lathhouse, and forcing conditions in a greenhouse. The growth chambers were Percival growth chambers (Model PGC - 105X). Photoperiods in growth chambers were set to match ambient photoperiods. Growth chambers were lit with a combination of 25-W incandescent and 160-W florescent bulbs (Phillips F27T12/CW/VHO)*.*

Starting January 31st 2017, we began to check for reproductive budburst on the cuttings twice weekly. We defined the day of year (DOY) of reproductive budburst as the first day we observed open male (staminate) catkins that were shedding pollen, or female (pistillate) catkins with bracts that had opened enough to allow for pollination. We also monitored the sampled trees from Webster Nursery for reproductive budburst from February 1st 2017 through March 20th 2017.

### Statistical analyses

To examine if relatively colder temperatures over the dormant season led to earlier dates of reproductive budburst than warmer temperatures, we compared the dates of reproductive budburst of red alder cuttings in treatments that experienced different dormant season temperatures, but then experienced the same forcing temperatures when moved to the greenhouse on January 31st (4 °C, 9 °C and Ambient/greenhouse treatments, Table 1). We statistically compared dates of budburst between the different treatments using a linear mixed-effects model with day of year (DOY) of budburst as the response variable and treatment as the predictor variable. We also initially included sex as a predictor variable in models to observe if there were differences in the timing of male and female budburst, and if the experimental treatments influenced those differences. The sampled tree ID was included as a random effect to account for male and female budburst observations from the same cutting, and to reduce the influence of variation between individual trees on final results. The *p*-values for comparisons between the three treatments were adjusted with a Bonferroni correction.

To examine whether the phenology of cuttings differed from phenology of the branches still attached to trees, we compared the DOY of reproductive budburst of cuttings in the lathhouse, which received ambient temperature conditions, to the originally sampled trees at Webster Nursery. We used a linear mixed-effects model with the DOY of reproductive budburst as the response variable, treatment (Ambient or Webster) and sex as predictor variables, and tree ID as a random variable. All models were conducted using the lmer function in the lmertest package (Kuznetsova, Brockhoff & Christensen, 2017) in the statistical program R (R Core Team, 2017). Code and data for the analyses and are included in the Files S2–S4.

### Testing the reproductive phenology model

We used the reproductive phenology model described in Prevéy, Harrington & St. Clair (2018) to calculate the chilling and forcing hour accumulations by the date of reproductive budburst of cuttings in all treatments. The model modified functions from a previous model of vegetative budburst (Harrington, Gould & St.Clair, 2010) to give effectiveness units to hourly temperatures over the dormant season that vary from 0 to 1 (Fig. S1). The chilling and forcing units are then summed from November 1st through the date of budburst and a “possibility line” is fit to the data. The possibility line shows all the different combinations of chilling and forcing unit accumulations that can result in reproductive budburst (Prevéy, Harrington & St. Clair, 2018). The reproductive phenology model was originally parameterized using a large database of Douglas-fir flowering data, which allowed for the determination of a possibility-line for flowering of Douglas-fir (Prevéy, Harrington & St. Clair, 2018). Here, we test whether the equations developed to estimate the effectiveness of chilling and forcing temperatures for flowering of Douglas-fir (Fig. S1) could be used to estimate a possibility line for reproductive budburst of red alder. We calculated hourly chilling and forcing units, and summed unit accumulations by the date of reproductive budburst, for cuttings from all experimental treatments as well as for reproductive budburst dates from trees at Webster Nursery. We then fit the possibility-line with a hierarchical linear model to account for variation in the date of reproductive budburst between different treatments and individual cuttings. We fit both linear and logarithmic models and compared fit statistics to identify the best-fit possibility line for reproductive budburst of red alder.

## Results

**Figure 1:**
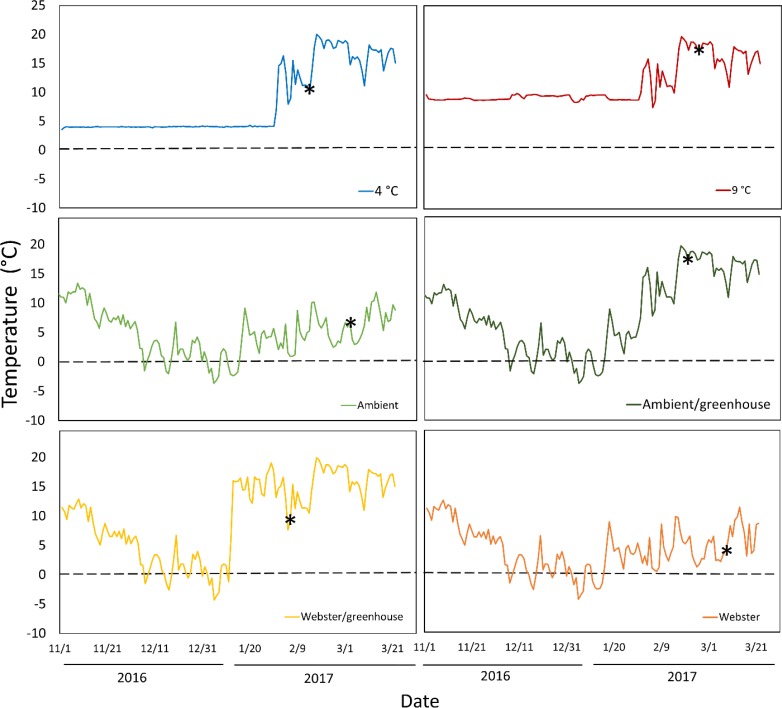
Daily mean temperatures for all experimental treatments over the course of the experiment: November 2nd 2016–March 26th 2017. Asterisks indicate the date of mean reproductive budburst for red alder cuttings in each treatment. The dashed line denotes 0 °C.

Temperature conditions averaged 4 °C in the colder growth chamber, and 9 °C in the warmer growth chamber (Fig. 1). Ambient conditions in the lathhouse and at Webster Nursery averaged 4.4 °C, and 4.3 °C, respectively, from November 2nd 2016–January 31st 3017, and there were multiple freezing events (Fig. 1).

Across the treatments, an average of 63% of red alder cuttings survived to reproductive budburst (Table 2). The reproductive buds on one of marked branches of the ten originally sampled trees at Webster Nursery stopped developing in mid-winter and did not flower in spring (Table 2).

**Figure 2:**
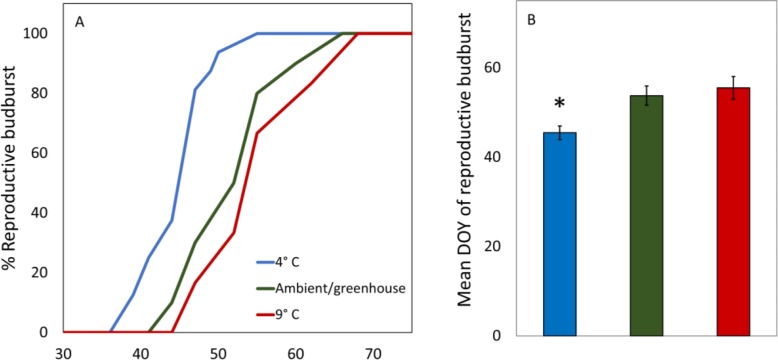
Average DOY of reproductive budburst for red alder cuttings in the 4 °C, 9 °C, and ambient/greenhouse treatments that all received the same forcing temperatures in the greenhouse from January 31st 2017 onward. An asterisk above a bar denotes significant differences at the *p* < 0.05 level.

There were no significant differences in the dates of reproductive budburst between male and female catkins in the 4 °C treatment (*t* =  − 0.72, *p* = 0.48, *df* = 19.59, Table 2). The 9 °C and Ambient/greenhouse treatment had lower survival and thus a statistical test of differences between the dates of male and female budburst was not possible. Red alder cuttings in the 4 °C treatment had earlier male and female reproductive budburst than red alder cuttings in the 9 °C or ambient/greenhouse treatments (*t* > 2.1, *p* < 0.002, *df* = 26.33, Table 2, Figs. 2A and 2B). Cuttings in the warm treatment took the longest to reach 100% reproductive budburst (Fig. 2A).

**Table 2:** Number of cuttings with male or female reproductive buds that survived to budburst, and the average DOY of reproductive budburst for each sex in experimental treatments and at Webster Nursery over the course of the experiment (11/2/2016 –3/26/2017). Bolded values indicate significant (*p* < 0.05) differences between the day of year (DOY) of male and female budburst within a treatment.

Treatment	# of surviving cuttings	Sex	# of cuttings with reproductive buds	DOY flowering +/-SE
4 °C	9	M	9	44.8 ± 1.4
		F	7	46.9 ± 1.6
9 °C	5	M	4	55.7 ± 2.3
		F	4	57.3 ± 6.3
Ambient	5	M	5	64.5 ± 3.2
		F	0	N/A
Ambient/greenhouse	5	M	4	53.2 ± 2.7
		F	4	53.5 ± 3.1
**Webster**	**9**	**M**	**9**	**67.5 ± 4.2**
		**F**	**9**	**72.8 ± 0.9**
**Webster/greenhouse**	**6**	**M**	**6**	**34 ± 2.0**
		**F**	**4**	**45.5 ± 5.0**

All red alder twigs had both male and female catkins, however, after male reproductive budburst, female catkins in the ambient treatment did not develop further (Table 2). We found no significant differences between the timing of male budburst of cuttings in the Ambient treatment compared to budburst of marked branches at Webster Nursery (*t* = 0.76, *p* = 0.46, *df* = 17.99, Table 2). There was earlier reproductive budburst for male versus female red alder cuttings harvested on January 11th 2017 in the Webster/greenhouse treatment, and on trees at Webster Nursery (Table 2).

### Reproductive phenology models

**Figure 3:**
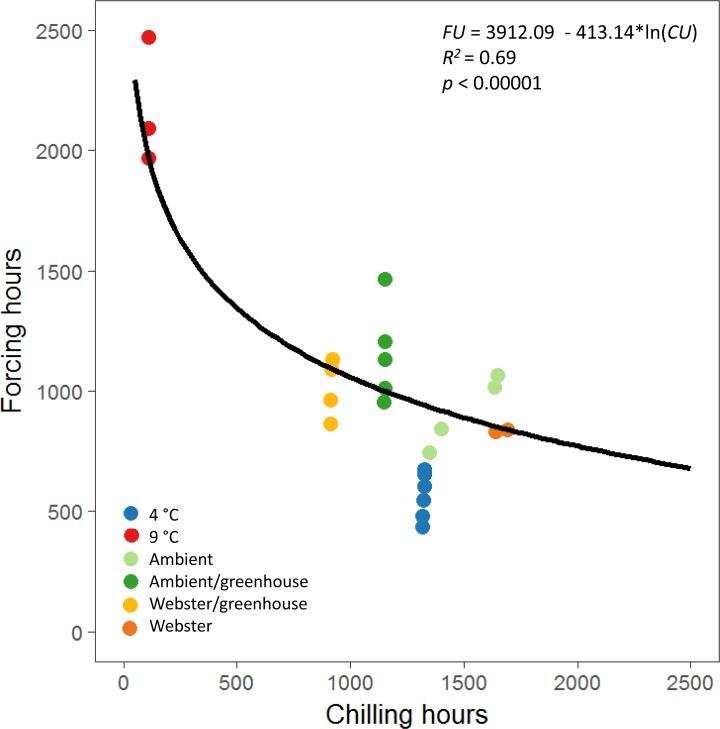
Natural log relationship between chilling and forcing accumulations for red alder. The solid black line shows the slope of the relationship between chilling units accumulated by reproductive budburst and forcing units accumulated by reproductive budburst across the different experimental treatments. On average, points above the line indicate combinations of chilling and forcing where reproductive budburst is likely, and points below the line indicate combinations where budburst is less likely to occur.

The equations for the reproductive phenology model in Prevéy, Harrington & St. Clair (2018) were used to define a possibility-line for reproductive budburst of red alder (Fig. 3). We used only male reproductive budburst data to model the possibility-line, as we had more observations of male reproductive budburst than female reproductive budburst. A natural log relationship between chilling and forcing unit accumulation fit the data better than a linear relationship (*R*^2^ = 0.69 for the natural log model versus *R*^2^ = 0.49 for the linear model, Fig. 3).

## Discussion

We found that pretreatment with colder (4 °C) winter temperatures accelerated the reproductive budburst of red alder more than warmer (9 °C) winter temperatures after cuttings were placed in forcing conditions in a greenhouse. These results provide preliminary evidence that relatively colder temperatures may be more effective for satisfying chilling requirements for reproductive budburst of temperate tree species that flower prior to leaf-out in spring. The importance of cold temperatures for flowering also suggests that warmer winter temperatures in the future may not be as effective for satisfying chilling requirements for flowering trees (Luedeling, 2012). Current mean winter (Dec.–Mar.) temperatures across the range of red alder vary from 8.6 °C along the southern range limits in California to −5.2 °C at the northern range limits in Alaska (Wang et al., 2016). Mean winter temperatures are projected to increase from 4 to 8 °C by 2080 (Wang et al., 2016). These appreciable temperature increases, especially along the southern portion of the species range, could eventually lead to a delay in reproductive budburst (Luedeling, Zhang & Girvetz, 2009).

Although colder temperatures led to earlier reproductive budburst, cuttings from all temperature treatments in this experiment did experience male reproductive budburst, indicating that a fairly wide range of winter temperatures (4 to 9 °C) can contribute to chilling requirements. A wide range of temperatures was similarly found to be effective for chilling prior to vegetative budburst of Douglas-fir (Harrington, Gould & St.Clair, 2010). Additionally, we found that increased exposure to chilling temperatures led to less forcing temperatures required prior to reproductive budburst, similar to other studies of vegetative and reproductive phenology (Harrington, Gould & St.Clair, 2010; Nanninga et al., 2017; Prevéy, Harrington & St. Clair, 2018). Thus, even if winter temperatures become warmer in the future, our results indicate that increasing temperatures in spring may still result in advancing budburst dates.

The equations used to calculate chilling and forcing unit accumulations for the reproductive phenology model of Douglas-fir (Prevéy, Harrington & St. Clair, 2018) worked well to describe a possibility-line for reproductive budburst of red alder. The best fit model for the Douglas-fir possibility-line was linear, whereas a log model was a better fit for the experimental red alder flowering data, which covered a wider range of winter temperatures than the observational data used to create the Douglas-fir model (Prevéy, Harrington & St. Clair, 2018). Examining how phenology is altered under a wide range of experimental temperatures is important, as it can be difficult to predict the effects of novel climates from observational field data if the field data doesn’t include the range of temperature conditions that may occur in the future (Harrington, Gould & St.Clair, 2010).

Our test to observe whether the phenology of cuttings in the ambient treatment was a good proxy for phenology on whole trees was met with mixed results. On one hand, the timing of male reproductive budburst in the ambient treatment was very similar to the timing of male reproductive budburst outside on trees. This indicates that the reproductive phenology of cuttings can match that of branches on intact trees, and can be a useful way to expose reproductive buds to experimental conditions (Vitasse & Basler, 2014). On the other hand, the development of all female reproductive buds stopped prior to budburst on the cuttings in the ambient treatment, whereas most female reproductive buds on trees continued to develop. So, buds on cuttings may not develop in the same way as those on trees, especially if they are removed from trees for long time periods. Perhaps shortening the length of time cuttings are kept in growth chambers, or adding nutrients to the water that cuttings are kept in, may result in more female reproductive budburst of cuttings. Future experiments with a high replication of cuttings within treatments (to account for mortality over the course of the experiment), as well as diverse genotypes from across the range of tree species would allow for more robust examinations of how environment and adaptation influence the timing of reproductive budburst.

We observed much earlier reproductive budburst in our experimental treatments than was observed for red alder in outside conditions. While we did not specifically alter photoperiod in this study, our results indicate that the influence of temperature alone can accelerate reproductive budburst much earlier than has happened historically, indicating that photoperiod may not constrain the advancement of early-season phenology of trees in the Pacific Northwest. However, future research using reproductive cuttings should include treatment combinations that alter both photoperiod and temperature, as there may be interactive effects between temperature and photoperiod (Heide, 1993; Basler & Körner, 2012) that may influence phenological responses to climate change (Laube et al., 2014; Way & Montgomery, 2015).

## Conclusion

This experiment provides evidence that warmer winters with climate change may not be as effective as current conditions for satisfying chilling requirements of reproductive budburst of red alder. However, multiple different combinations of chilling and forcing temperatures can result in reproductive budburst of red alder, similar to vegetative budburst of other Pacific Northwest tree species (Harrington & Gould, 2015). These results provide information on the effectiveness of different temperatures for chilling requirements prior to red alder reproductive budburst. This information can then be used to predict how the timing of reproductive budburst may change in the future.

##  Supplemental Information

10.7717/peerj.5221/supp-1 Supplemental Information 1 Supplemental Information and code S1. Chilling and forcing functions used to estimate the ”possibility-line” for reproductive budburst of red alder.
S2. Code for all analyses in manuscript. Code was run in the statistical program R (R Core Team, 2017).

10.7717/peerj.5221/supp-2 Supplemental Information 2 Raw data: dates of reproductive budburst of red alder cuttings in all treatments

10.7717/peerj.5221/supp-3 Supplemental Information 3 Raw data: temperature data for all treatments from 1/2/2016 through 4/6/2017

## Additional Information and Declarations

### Data Availability

The following information was supplied regarding data availability: The raw data and code are available as Supplemental Files.
