# Supplementary material for: Effectiveness of winter temperatures for satisfying chilling requirements for reproductive budburst of red alder (Alnus rubra)
Source: PeerJ. 2018 Sep 25;6:e5221. doi: 10.7717/peerj.5221 (PMC6161696; doi:10.7717/peerj.5221)
Supplement: Supplemental Information 1 — S1. Chilling and forcing functions used to estimate the ”possibility-line” for reproductive budburst of red alder. S2. Code for all analyses in manuscript. Code was run in the statistical program R (R Core Team, 2017). [file peerj-06-5221-s001.docx]

Supplement.

**Fig S1**. Chilling and forcing functions from Prevéy et al (2018).

**S2.** Code for all analyses in manuscript. Code was run in the statistical program R (R Core Team 2017).

##load packages used for analyses and graphing

library(plyr)

library(ggplot2)

library(gridExtra)

library(lmerTest)

##### Compare DOY of budburst between sexes and treatments #########################

dat <- read.csv("S3.csv", header=T)

### Examine differences in timing of budbust of treatments experiencing the same forcing temepratures

## remove other treatments

Al <-dat[!dat$Treatment=='Ambient',]

Al <-Al[!Al$Treatment=='Webster/greenhouse',]

Al <-Al[!Al$Treatment=='Webster',]

hist(Al$DOY)

modl <- lmer(data=Al, DOY ~ Treatment*Sex + (1|ID))

summary(modl)

modl <- lmer(data=Al, DOY ~ Treatment + Sex + (1|ID))

summary(modl)

### remove sex as it is not a sig. predictor

modl <- lmer(data=Al, DOY ~ Treatment + (1|ID))

summary(modl)

## reorder factors

Al$Treatment <- relevel(Al$Treatment, ref = "9")

summary(modl)

### adjust p values for multiple comparisons

pvals <- c(0.000694,0.000561,0.557363)

p.adjust(pvals, method = "bonferroni")

### Examine differences in timing of budbust of branches on intact trees versus cuttings experiencing ambient conditions

## remove other treatments

amb <-dat[!dat$Treatment=='Ambient/greenhouse',]

amb <-amb[!amb$Treatment=='Webster/greenhouse',]

amb <-amb[!amb$Treatment=='4',]

amb <-amb[!amb$Treatment=='9',]

## limit to male reproductive budburst, as female buds did not open on cuttings in lathhouse

amb <-amb[!amb$Sex=='F',]

modl <- lmer(data=amb, DOY ~ Treatment + (1|ID))

summary(modl)

###Functions for calculating reproductive budburst from Prevey et al (2018)##

############################################################################

### hourly temperature measurements

temp.df <- read.csv("S4.csv", header=T)

## function for calculating daily chilling units

FunChill <- function(TEMP, aC, bC, cC, dC, eC){

c <- aC * (((TEMP + bC)/cC)^dC) * exp(-(((TEMP + bC)/cC)^eC))

c <- ifelse(c > 1, 1, c)

c <- ifelse(TEMP <= - 20 | TEMP >= 16, 0, c)

return(c)

}

aC <- 3.13; bC <- 10; cC <- 10.93; dC <- 2.10; eC <- 3.10

## function for calculating daily forcing units

FunForce <- function(TEMP, aF, bF){

f <- 1 / (1 + exp(aF + bF*TEMP))

return(f)

}

aF <- 4.49; bF <- -0.63

## calculate all forcing and chilling units for each day

temp.df$CU <-FunChill(temp.df$temp, aC, bC, cC, dC, eC)

temp.df$FU <-FunForce(temp.df$temp, aF, bF)

#### replace NAs with 0

temp.df$CU[is.na(temp.df$CU)] <- 0

temp.df$FU[is.na(temp.df$FU)] <- 0

## accumulate chilling and forcing units to flowering date

temp.df$FUsum <- ave(temp.df$FU,temp.df$treatment, FUN=cumsum)

temp.df$CUsum <- ave(temp.df$CU,temp.df$treatment, FUN=cumsum)

temp.df$treatday<- paste(temp.df$treatment,temp.df$DOY, temp.df$hour)

############ merge hourly accumulations with all flowering dates used for model fit #########

### limit to male reproductive budburst as no female budburst occurred in lathhouse

fit.dates <-alder[!alder$Sex=='F', ]

fit.dates$treatday <-paste(fit.dates$Treatment, fit.dates$DOY,fit.dates$hour )

## merge flowering dates and chilling/focring calculations to get accumulations by DOY of budburst

preddata <- merge(temp.df,fit.dates, by = "treatday")

## remove low survival freeze treatment before making possibility line

preddata <-preddata[!preddata$Treatment=='4/freeze', ]

### calculate possibility line for reproductive budburst #########################################################

line1 <- lmer(data=preddata, FUsum ~ (CUsum) + (1|ID))

line2 <- lmer(data=preddata, FUsum ~ log(CUsum) + (1|ID))

summary(line1)

summary(line2)

AIC(line1,line2)

#### compare R squared values of linear fits

line1 <- lm(data=preddata, FUsum ~ (CUsum))

line2 <- lm(data=preddata, FUsum ~ log(CUsum))

summary(line1)

summary(line2)

#### pseudo R square function ###

r2.corr.mer <- function(m) {

lmfit <- lm(model.response(model.frame(m)) ~ fitted(m))

summary(lmfit)$r.squared

}

r2.corr.mer(line1)

r2.corr.mer(line2)

#### graph results

theme_set(theme_bw(base_size =20))

### possibility line from above model ###

mycurve <- function(x){ a + (b*log(x)) }

a <- 3912.091

b <- -413.143

###############Alder chilling and forcing graph ####################

bam <- ggplot(preddata, aes(x=CUsum, y=FUsum, color=Treatment)) +

geom_point(size = 5) + scale_x_continuous(name= "Chilling hours", limits = c(0,2500)) + scale_y_continuous(name= "Forcing hours", limits = c(0,2500))

d <- bam + stat_function(fun=mycurve, color="black", size = 2)

man <- d + ggtitle("Chilling and forcing accumulations for Alder") + theme(panel.grid.major=element_blank(),panel.grid.minor=element_blank())

legend.title <- "Treatment"

man + scale_color_manual(legend.title, values=c("#1f78b4", #cold

"#e31a1c", # warm

"#b2df8a", #lathhouse

"#33a02c", #lathhouse_greenhouse

"darkgoldenrod1", #web_out

"chocolate2" ##web_green

))
